# A Fecal-Microbial-Extracellular-Vesicles-Based Metabolomics Machine Learning Framework and Biomarker Discovery for Predicting Colorectal Cancer Patients

**DOI:** 10.3390/metabo13050589

**Published:** 2023-04-25

**Authors:** Fatma Hilal Yagin, Abedalrhman Alkhateeb, Cemil Colak, Mohammad Azzeh, Burak Yagin, Luis Rueda

**Affiliations:** 1 Department of Biostatistics and Medical Informatics, Faculty of Medicine, Inonu University, 44280 Malatya, Turkey; hilal.yagin@inonu.edu.tr (F.H.Y.); cemil.colak@inonu.edu.tr (C.C.); burak.yagin@inonu.edu.tr (B.Y.); 2 Software Engineering Department, King Hussein School of Computing Science, Princess Sumaya University for Technology, Amman P.O. Box 1438, Jordan; 3 Data Science Department, King Hussein School of Computing Science, Princess Sumaya University for Technology, Amman P.O. Box 1438, Jordan; m.azzeh@psut.edu.jo; 4 School of Computer Science, University of Windsor, Windsor, ON N9B 3P4, Canada

**Keywords:** colorectal cancer, metabolomics profiling, machine learning, biomarker discovery

## Abstract

Colorectal cancer (CRC) is one of the most common and lethal diseases among all types of cancer, and metabolites play a significant role in the development of this complex disease. This study aimed to identify potential biomarkers and targets in the diagnosis and treatment of CRC using high-throughput metabolomics. Metabolite data extracted from the feces of CRC patients and healthy volunteers were normalized with the median normalization and Pareto scale for multivariate analysis. Univariate ROC analysis, the *t*-test, and analysis of fold changes (FCs) were applied to identify biomarker candidate metabolites in CRC patients. Only metabolites that overlapped the two different statistical approaches (false-discovery-rate-corrected *p*-value < 0.05 and AUC > 0.70) were considered in the further analysis. Multivariate analysis was performed with biomarker candidate metabolites based on linear support vector machines (SVM), partial least squares discrimination analysis (PLS-DA), and random forests (RF). The model identified five biomarker candidate metabolites that were significantly and differently expressed (adjusted *p*-value < 0.05) in CRC patients compared to healthy controls. The metabolites were succinic acid, aminoisobutyric acid, butyric acid, isoleucine, and leucine. Aminoisobutyric acid was the metabolite with the highest discriminatory potential in CRC, with an AUC equal to 0.806 (95% CI = 0.700–0.897), and was down-regulated in CRC patients. The SVM model showed the most substantial discrimination capacity for the five metabolites selected in the CRC screening, with an AUC of 0.985 (95% CI: 0.94–1).

## 1. Introduction

Colorectal cancer (CRC) is the third most common cancer and second in terms of mortality among all cancers for both sexes [1]. Genetic, environmental, and lifestyle factors have been found to be possible causes of CRC. While heredity plays a crucial role in the development of the disease, most CRC cases are sporadic [2], and these cases are observed at an increasing rate [3]. In cancer, there is a significant shift in the metabolic processes that sustain normal cells. These changes, called metabolic reprogramming, play critical roles in the initiation and progression of cancer in general [4], and CRC is no exception [2].

Biomarkers are biological indicators that can be objectively measured and used to diagnose, monitor, or predict disease risk. Atikukke et al. studied gene mutations in a cohort of early-onset biomarkers. They stated that malignancies appear to be microsatellite-stable (MSS) with a minor tumor mutation burden according to the tumor mutational burden (TMB) measure for the genomic profiling data of these patients’ initial samples. With a notable mutation frequency of PIK3R1, PDGFRA, FLT3, and KDR gene alterations, this group of individuals appears to have a different mutational landscape [3]. However, the main problem of genomic-based approaches is the invasive nature of the biopsy, which is usually accompanied by pain and bleeding.

Metabolomics analysis may provide potential advantages through the discovery of a suite of non-invasive, clinically relevant biomarkers that can identify CRC [5]. Brown et al. examined the relationship between colorectal cancer and metabolic dysfunction. Their work focused on understanding the molecular and cellular mechanisms underlying the connection between metabolic disorders and the development of colorectal cancer. The findings suggest that metabolic alterations can lead to the activation of oncogenic signaling pathways, promotion of oxidative stress, and suppression of the immune response, all of which contribute to the development and progression of colorectal cancer [6]. To establish a strategic treatment protocol for CRC, Khan et al. investigated the protein expression in a mouse model with different levels of radio responsiveness. The model extracted nine differentially expressed proteins, namely, PGK1, PGAM1, ENO1, PKM, TKT, GLUD1, LDHA, GAPDH, and MDH2 [7]. Machine learning approaches have been applied to metabolites to identify biomarkers for CRC [8,9]. Kim et al. performed a combination of metabolic analysis and microbiome profiling of extracellular vesicles (EVs) obtained from the stools of CRC patients compared to healthy volunteers. The model applied multivariate and univariate analyses to the metabolomics data using Metaboanalyst 4.0. The dataset was normalized using log transformation, and the Pareto scaling was analyzed. Then, principal component analysis (PCA) was applied to examine the differentiation in the metabolic profiles between the healthy control group and the CRC patient group. Univariate analysis, using false-discovery-rate (FDR)-adjusted *p*-values, was used for the selected metabolic candidates in the case of both classes. Significant differences were determined using the Wilcoxon test for continuous variables. Findings were considered significant if the *p*-value < 0.05. The results suggest possible correlations between the metabolism of gut microbes and the changes in the identified metabolites according to the pathophysiology of the disease [8]. Hossain et al. applied a machine learning model to investigate CRC transcriptome data in order to identify associations between disease relationships and CRC survival. The authors utilized protein–protein interaction (PPI) results, gene expression analysis, and clinical data to identify a signature for different CRC classes. CRC samples from tissues were exposed to the following eight risk factors: aging (AG), type II diabetes (T2D), high consumption of alcohol (AC), obesity (OB), high-fat diet (HFD), high intake of red meat (RM), long-term smoking (SM), and a high-calorie diet (HCD). These datasets were analyzed and cross-compared to identify overlapping, differentially expressed genes (DEGs) that could potentially serve as biomarkers for CRC [9].

It has recently come to light that microbe-derived extracellular vesicles, often known as EVs, are becoming an essential new research subject in the quest to understand the relationship between the gut microbiota and human health. Different kinds of EVs are capable of being secreted by gut microbiota, including outer-membrane vesicles (OMVs), shedding vesicles, and apoptotic bodies. EVs are made up of lipids, proteins, nucleic acids, and metabolites for the most part. Their principal function is to carry active biomolecules to cells over long distances, facilitating medication delivery to specific areas or modulating host cellular responses. Although the underlying mechanisms are still not fully understood, this is their fundamental task [8,10,11,12,13,14]. Recent research has provided some evidence showing that the gut microbiome plays a role in the development of CRC [14]. On the other hand, it is not yet known which metabolomics signals that are produced by bacteria in the gut lead to CRC.

This study aimed to investigate the biomarkers of CRC and develop a predictive model that can distinguish CRC patients by applying a methodology combining bioinformatics and machine learning approaches to metabolomics data, including the profile of metabolites in EVs from CRC patients versus healthy controls without known types of “omics” due to the nature of the model. This model conducts high-performance measurements with fewer gene biomarkers, whose number is 5 compared to the 15 gene biomarkers resulting from the work of Kim et al. [8].

## 2. Material and Methods

### 2.1. Study Design, Data, and Compliance with Ethical Standards

The data used in this study are available on the NIH Joint Fund’s National Metabolomics Data Repository (NMDR) website, Metabolomics Workbench (www.metabolomicsworkbench.org (accessed on 11 March 2023), where the project ID is designated as PR000888. The data can be accessed directly using the project DOI: 10.21228/M8WX1N. A total of 36 patients with colorectal cancer and 40 healthy patients as controls participated in this study. The characteristics of the patients, such as their age, gender, stage, tumor location, and carcinoembryonic antigen (CEA) test results, were evaluated. Healthy controls with no known disease and routine laboratory test results were included in the study. The exclusion criteria for healthy controls included a diagnosis of bowel disease, medication for bowel disease, and a previous diagnosis of CRC. General characteristics such as age, gender, and medical history were recorded for the healthy control subjects. The patient and healthy subject exclusion criteria were postoperative colorectal cancer recurrence, chemotherapy, complications of colorectal cancer with other cancers or metabolic diseases, drug therapy, or antibiotic therapy within one month of sample collection. The Metabolomics Standards Initiative (MSI) for all the included metabolites was set as 2. The metadata of the analyzed metabolites are listed in Table S1 in the Supplementary Materials. Table S2 shows the results of the permutation test for leucine and oxalic, with an accuracy of 0.87 for the test set.

The sample size required for this study was estimated with MetSizeR based on the PPCA model and calculated by setting the false discovery rate to 0.05. As a result, a minimum sample size of 14 patients in total with 7 patients in each group was estimated. Despite the difficulty of recruiting CRC patients and healthy controls who met the inclusion criteria defined in this study, the sample size exceeded the estimate obtained using MetSizeR [15], a method used to determine sample size in metabolomics studies. This study received ethical approval from the Inonu University Non-Interventional Clinical Research Institutional Review Board (decision no: 2022/4092). Informed consent was obtained from all subjects involved in the study.

### 2.2. Microbe-Derived Extracellular Vesicles’ Isolation and Gas Chromatography Time-of-Flight Mass Spectrometry Analysis

Stool samples were collected from the patients before surgery or bowel preparation. All individuals ate a light diet and did not smoke or drink alcohol the day before sample collection. A sample was taken from the stool center of each patient using a sterile cotton swab and stored at −20 °C. Afterward, the samples were incubated to separate the microbe-derived EVs from the human feces. After thawing the frozen EV samples obtained from all the patients, metabolomic analysis was performed using gas chromatography time-of-flight mass spectrometry (LC/QTOFMS) and gas chromatography-TOFMS (GC/TOFMS).

### 2.3. Data Analysis

**Figure 1:**
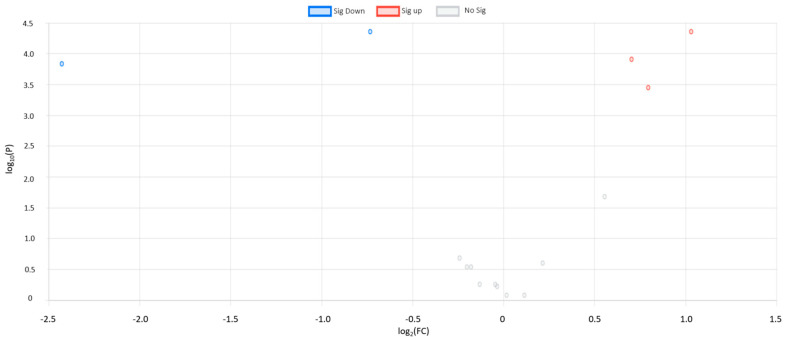
Volcano plot that shows statistical significance (*p*-value) versus magnitude of change (fold change) for the identified metabolites.

The metabolomic data were analyzed using univariate and multivariate statistical methods. The data were normalized using the median and Pareto-scaled for multivariate analysis. Significant changes in metabolite levels were tested using the *t*-test, and FDRs were determined according to the Benjamini–Hochberg procedure to minimize the false positives. Fold changes (FCs) were calculated to examine the differences between the metabolites from the CRC patients and those from the healthy patients. FDR-adjusted *p*-values < 0.05 and FCs ≥1.5 (up-regulated) or ≤1.5 (down-regulated) were considered significant. Furthermore, we visualized the metabolites that were consistently up- or down-regulated in the CRC patients compared to the healthy controls with a Volcano plot for exploratory biomarker analysis, as depicted in Figure 1. To identify the metabolic signature contributing to group discrimination and to evaluate the predictive performance of potential biomarkers in distinguishing CRC, separate or combined ROC curve analysis was performed. The results are presented with the 95% confidence interval (CI) for the AUC with the ROC curve. The ROC curves were produced using the balanced subsampling technique known as Monte Carlo cross-validation (MCCV). Two-thirds (2/3) of the samples in each MCCV were used to determine the feature’s importance. In this context, the features are the metabolites genes, and the importance of the feature determines the features with the most discriminative features between the two classes. The most important features were used to build classification models, which were then tested on one-third of the excluded samples [16]. To increase the accuracy and robustness of our analysis, we combined the results of the *t*-test and the FC, as well as those of the *t*-test and ROC analysis, to identify reliable biomarker candidate metabolites that significantly contributed to the differentiation of the CRC and healthy patients. Therefore, we considered only metabolites overlapping the two different statistical approaches for further analysis (FDR-corrected *p*-value < 0.05 and AUC > 0.70). Multivariate analyses were performed using the ROC curve method with biomarker candidate metabolites based on linear support vector machine (SVM) [17], partial least squares discrimination analysis (PLS-DA) [18], and random forest (RF) [19] algorithms. These methods have proved to be robust for high-dimensional data and are widely used for other types of ‘omics’ data analysis. The area under the curve (AUC with 95% CI), sensitivity, and specificity were calculated to estimate the performance of the models.

## 3. Results

### 3.1. Univariate Statistical Analysis

The univariate analyses revealed five biomarker candidate metabolites that differed significantly (adjusted *p*-value < 0.05) in the CRC patients compared to the healthy controls. Our results showed that aminoisobutyric acid and butyric acid were down-regulated in the CRC patients, while succinic acid, isoleucine, and leucine were up-regulated. When the FC values were examined, aminoisobutyric acid and butyric acid were down-regulated 0.60- and 0.18-fold in the CRC patients compared to the healthy controls. In addition, succinic acid, isoleucine, and leucine were up-regulated 2.04-, 1.63-, and 1.73-fold in the CRC patients, respectively (Table 1 and Figure 1). Figure 1 depicts the volcano plot for the five significantly expressed metabolites. It is clearly noticeable that the value of butyric acid is negatively folded twice with Log_2_ FC, being −2.43.

### 3.2. Biomarker Analysis

**Figure 2:**
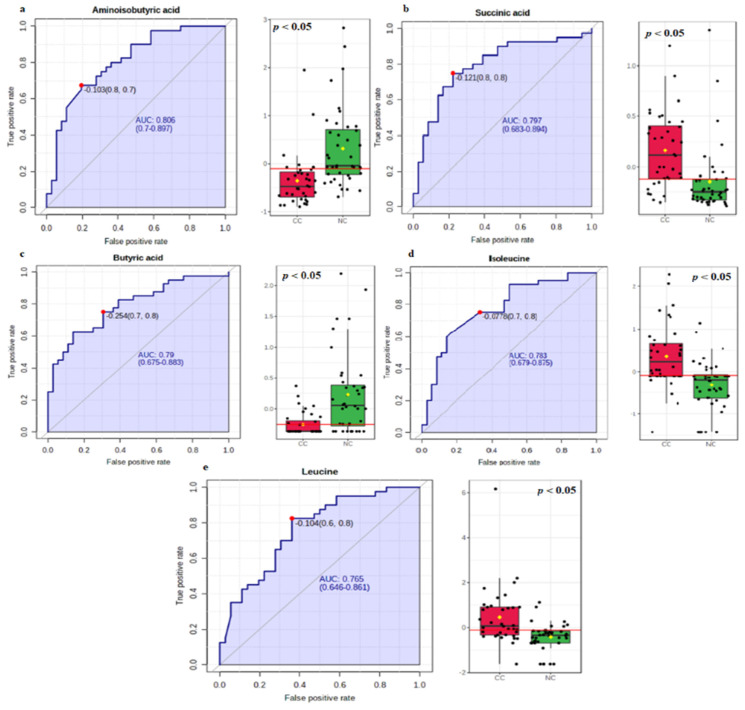
ROC curve analysis was performed separately for each of the five metabolically predicted potential biomarker candidates in the CRC (red) and healthy control (green) groups. ROC analysis (**left** panel) and box-whisker plot (**right** panel) for (**a**) aminoisobutyric acid, (**b**) succinic acid, (**c**) butyric acid, (**d**) isoleucine, and (**e**) leucine. The box-whisker plots revealed that the aminoisobutyric acid and butyric acid levels were significantly decreased and the succinic acid, isoleucine, and leucine levels were significantly increased in CRC patients compared with the healthy control (*p* < 0.05).

In the first part of the analysis, we analyzed the metabolic processes involved in the pathophysiology of CRC to better understand the metabolic heterogeneity of CRC. In the second part, we performed exploratory biomarker analysis to identify biomarkers that could distinguish CRC from the healthy controls. To test the utility of the metabolic profiles in the diagnostic screening of CRC patients, we performed ROC curve analysis to evaluate the diagnostic potential of the metabolites in order to discriminate between the healthy controls and CRC patients. With an AUC value > 0.7 and *p*-value < 0.05 as the criteria for diagnostic potential, five metabolites (aminoisobutyric acid, butyric acid, succinic acid, isoleucine, and leucine) were identified as potential diagnostic biomarkers of CRC. The individual ROC curve analysis results are presented with the cut-off point, AUC with 95% CI, sensitivity, and specificity values that show how effectively the selected candidate biomarkers can discriminate between the two diagnostic groups (CRC/healthy control). Our results showed that aminoisobutyric acid had the highest discriminative potential, with an AUC equal to 0.806 (95% CI = 0.700–0.897), and leucine had the lowest discriminatory potential, with an AUC equivalent to 0.765 (95% CI = 0.646–0.861) (Table 2, and Figure 2). Figure 2 shows the individual prediction power of each metabolite, showing a relatively wide AUC range from 0.765 to 0.805.

### 3.3. Multivariate Analysis

**Figure 3:**
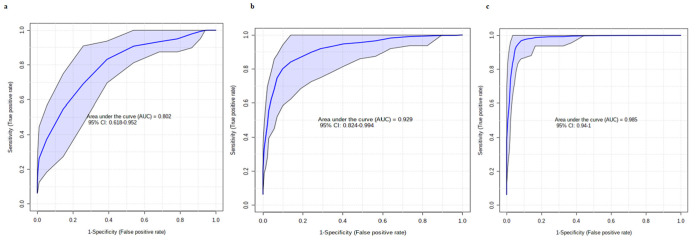
ROC curves based on cross-validation (CV) performance. (**a**) ROC graph for PLS-DA model. (**b**) ROC graph for the RF model. (**c**) ROC graph for the SVM model. The ROC curves are the curves of the models for the five biomarker candidate metabolites with the 95% confidence interval calculated for each model.

**Figure 4:**
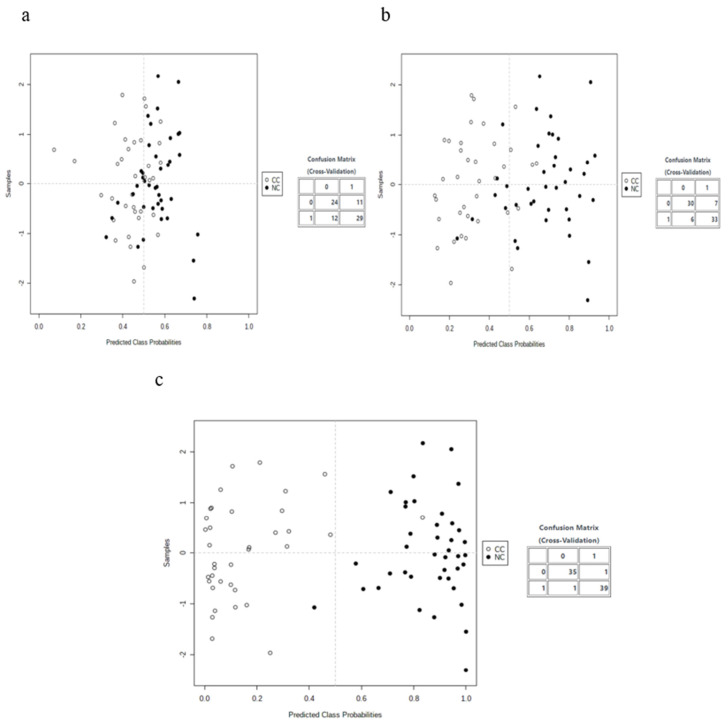
Estimated class probabilities of each sample with 10 CVs and the confusion matrix (CC or NC). (**a**) Results of the PLS-DA model. (**b**) Results of the RF model. (**c**) Results of the SVM model.

In the third part of the analysis, we combined five biomarker candidates and plotted the receiver operating characteristic (ROC) curves with the 95% CI to develop biomarker models based on machine learning and statistical algorithms (SVM, PLS-DA, and RF). A total of 10 cross-validations (CVs) were performed to generate the ROC curves, and the results were averaged to generate the plots. The SVM model with a 0.985 (95% CI: 0.94–1) AUC showed strong discrimination capacity for the five metabolites selected in the CRC screening compared to the PLS-DA, with a 0.802 (95% CI: 0.618–0.952) AUC, and the RF models, with a 0.929 (95% CI: 0.824–0.994) AUC (Figure 3). For each model, the mean of the predicted class probabilities of each sample over 10 CVs was determined, and the confusion matrix is summarized in Figure 4. The model based on the SVM algorithm obtained the best estimate, correctly classifying 35 of the 36 samples from CRC patients and 39 of the 40 healthy control samples.

## 4. Discussion

Colorectal cancer, which is a prevalent kind of cancer, is a malignant tumor. Surgery, chemotherapy, radiotherapy, targeted therapy, and other forms of treatment are currently available for patients diagnosed with colorectal cancer. However, less than 15% of patients will live for five years after being diagnosed with colorectal cancer. Approximately 40% of CRC patients will eventually relapse and develop recurrence or late metastases. To develop a personalized treatment plan, it is essential to first identify the biomarkers linked with CRC and then forecast which patients will develop the disease [20].

This study may guide future clinical metabolomics studies aiming to search for different combinations of metabolic features with more reliable and robust diagnostic screening to differentiate CRC cases. The findings of this work agree with the results of the work conducted by Kim et al., where succinic acid, isoleucine, and leucine were up-regulated, while aminoisobutyric acid was down-regulated. However, the predictability of Kim et al.’s model was 92.0% with 15 metabolic biomarkers [8], compared to the proposed model, which increased the performance, with a predictability of 98.5% and only 5 biomarkers. A small number of biomarkers with greater predictability can efficiently aid in the diagnosis of CRC. Aminoisobutyric and butyric (butanoic) acids are essential for modulating host metabolic and immune responses in the human intestine [8]. Increased succinic acid promotes tumor growth, including breast, lung, bladder, and colorectal cancer [21].

Terasaki et al. suggested that succinic acid is a prognostic biomarker for CRC. The results strongly indicate physiological changes in human colorectal cancer stem cells (CCSCs) induced by Fucoxanthinol (FxOH) treatment. Based on metabolite profiling via GC-MS analysis, the authors reported that reduced glycine and succinic acid levels were correlated with EMT suppression and apoptosis induction in human colorectal-cancer-stem-cell (CCSC)-like spheroids (colonospheres, Csps) [22]. Long et al. reported that isoleucine acid is linked to survival after diagnosis and is a prognostic biomarker for CRC. The model was used to evaluate the associations between post-diagnostic branched-chain amino acid (BCAA) intake with CRC-specific mortality and overall mortality among 1674 patients with nonmetastatic CRC in the Nurses’ Health Study and the Health Professionals Follow-up Study. Both isoleucine and leucine showed statistically significant associations with each of the BCAA intakes observed for CRC-specific mortality [23]. A study suggested that restricting the amount of leucine in the diet could benefit CRC patients [24]. Leucine plays the role of an anabolic signal for amino acid assembly into new proteins. Suryawan et al. showed that the acute (1 h) administration of leucine promotes muscle protein synthesis by activating translation initiation factors downstream of mTORC1. The model yielded an important result showing that KIC, but not norleucine, can replace leucine’s action, but the authors suggested that more investigation are needed to evaluate the chemical structures required for the leucine-induced stimulation of protein synthesis [24].

Multivariate analysis is based on multivariate statistics. Typically, it addresses situations where multiple measurements of each experimental unit are made, and the relationships between these measurements and their structures are important. It is complicated by the problem’s dimensionality, where the number of features is very high compared to the number of samples. However, it is a powerful technique that can be used to search for the best feature subset that can represent the entire dataset with a high prediction performance [25]. The linear SVM model outperformed the PLS-DA and RF models. These three classifiers are known to perform well in multivariate analysis. SVM was able to predict both classes in the MCCV cross-validation with a high performance based on the five selected metabolites’ features. Figure 4c shows the large separation between the centers of the two classes for the linear SVM model, and this means that future tested samples could fall within this space.

Interestingly, the selected genes were both able to predict the two classes with AUC ranges from 0.765 to 0.806, as seen in Figure 2. Combined, these metabolites could predict the classes nearly perfectly, with an AUC equal 0.985. The computational model and literature analysis confirmed the association between the proposed metabolites’ signature and CRC.

**Figure 5:**
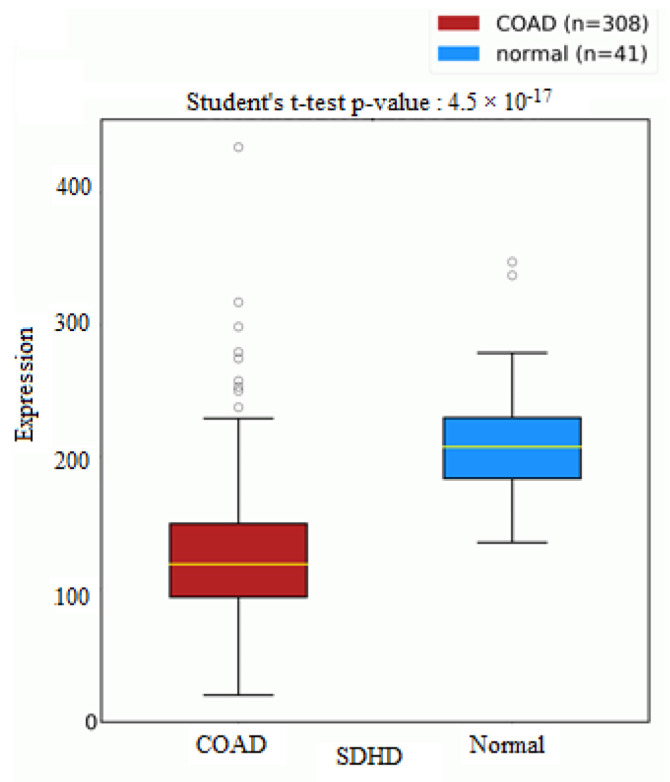
Boxplots for SDHD genes in colorectal cancer samples compared to normal control samples from the TCGA database. The plot was generated using oncoDB.org.

**Figure 6:**
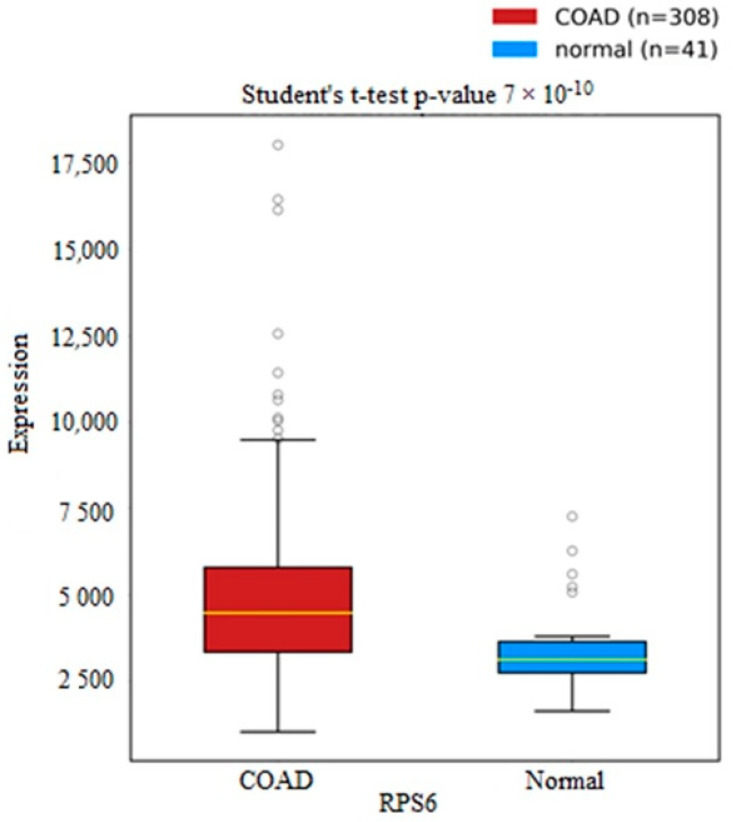
Boxplots for RPS6 genes in colorectal cancer samples compared to normal control samples from the TCGA database. The plot was generated using oncoDB.org.

The succinate dehydrogenase gene (SDH) is a gene associated with the production of succinic acid. Dysfunctions in succinate dehydrogenase (SDH) metabolic enzyme activity lead to an abnormal accumulation of succinic acid. SDHD is a subunit of the SDH gene that was found to be down-regulated in 308 colorectal cancer samples compared to 41 normal samples, as seen in Figure 5. Leucine acid is known to increase the rate of protein synthesis in skeletal muscle. Drummond et al. reported that leucine acid differentially regulates some mammalian targets of rapamycin complex 1 (mTORC1) proteins, including RPS6 [26]. RPS6 was found to be significantly up-regulated in TCGA samples compared to normal control samples, as seen in Figure 6.

In their mini-review, Zhang et al. reported that metabolites have a largely untapped potential for the diagnosis of CRC and oncology through the study of the cancer metabolome to identify metabolite biomarkers defined as surrogate indicators of physiological or pathophysiological states [5]. The research on metabolomics’ impacts on cancer, including the collection of samples, selection of samples, processing of samples, statistical analysis methods, and other such arduous tasks, is fraught with formidable difficulties. The absence of a secondary dataset that may be used to conduct an external validation of the model is the first limitation of this work. This limitation restricts the universality and applicability of the model. The second limitation of this study is that it was founded entirely on the abundance of metabolites in the patient tissues; alternative levels of clinical information or omics were not investigated. Therefore, further research combining clinical knowledge and different omics levels and examining their internal mechanisms of action in CRC is needed. Further validation of the findings may help to affirm the identified metabolites as biomarkers for CRC. The tumor microenvironment modulates cancer growth. Extracellular vesicles (EVs) serve as key mediators of intercellular communication [26]. Proteomics analysis of the findings may add further insight for understanding the production process of the amino acids in this disease.

## 5. Conclusions

This study explored a model that applies statistical and machine learning methods that extract metabolic biomarkers for CRC. The results suggest that five metabolites may serve as strong predictors of the disease, which may assist in diagnosis, progression, and treatment based on the measurement of these metabolites in the body. The metabolite biomarkers were extracted from the patients’ stools, which is a less invasive approach than tissue biopsy that leads to less pain and side effects. The findings were in agreement with Kim et al.’s [8] work using fewer metabolites, which is an excellent validation of the method. However, further validation may be required before applying the suggested biomarkers in practice.

The proposed machine learning model is based on multivariate feature analysis that selected five metabolites and incorporated them in an MCCV cross-validation to test three standard classifiers: linear SVM, PLS-DA, and RF. Linear SVM outperformed the other two by correctly classifying 35 of 36 samples from CRC patients and 39 of 40 healthy control samples with an accuracy of 97%.

The nature of this proposed machine learning model allows it to be used in other “omics” and for different health outcomes, including cancers and other complex diseases. The model may be used to integrate multi-omics data in order to better understand the disease progression. A future direction could be to study how the results of the model can be used in clinical practice, and wet-lab experiments could be conducted to validate the relationship between the extracted metabolites levels and the advancement of CRC cells. Integration of the findings with other omics and clinical data may help to explain how these omics work in the cell. Young sporadic CRC has been increasing in North America [3] in recent years, and applications of the proposed model to study the changes in metabolites may reveal more unknown information about these increasing numbers.

## Figures and Tables

**Table 1:** Univariate Analysis.

Metabolite Name	FC	Log_2_ FC	−Log_10_ (*p*-Value)	FDR Adjusted *p*-Value	Regulation	Main Class	Subclass
Succinic acid	2.04	1.03	4.35	8.64 × $10^{-4}$	UP	TCA acids	TCA acids
Aminoisobutyric acid	0.60	0.73	4.35	4.95 × $10^{-4}$	DOWN	Fatty acids	Amino fatty acids
Butyric acid	0.18	−2.43	4.17	4.60 × $10^{-4}$	DOWN	Fatty acids	Saturated fatty acids
Isoleucine	1.63	0.70	4.03	4.60 × $10^{-4}$	UP	Amino acids and peptides	Amino acids
Leucine	1.73	0.79	3.52	8.19 × $10^{-4}$	UP	Amino acids and peptides	Amino acids
Oxalic acid			1.55	0.07		Fatty acids	Dicarboxylic acids
Alanine			1.02	0.20		Amino acids and peptides	Amino acids
Ethanolamine			0.97	0.20		Amines	1,2-Aminoalcohols
Caproic acid			0.62	0.39		Fatty acids	Saturated fatty acids
Oleic acid			0.58	0.39		Fatty acids	Unsaturated fatty acids
Lysine			0.39	0.55		Amino acids and peptides	Amino acids
Phenol			0.33	0.58		Phenolic acids	Phenolic acids
2-Furoic acid			0.12	0.86		Furoic acids	Furoic acid derivatives
Palmitic acid			0.09	0.86		Fatty acids	Saturated fatty acids
Tyramine			0.04	0.91		Tyrosine alkaloids	Phenylethylamines

FC: fold change.

**Table 2:** Metabolites with good diagnostic value among CRC and healthy subjects were identified via biomarker analysis.

Metabolite Name	Cut-Off Point	AUC	95% CI	Sensitivity	Specificity
Aminoisobutyric acid	−0.103	0.806	0.700–0.897	0.675	0.805
Succinic acid	−0.121	0.797	0.683–0.894	0.750	0.770
Butyric acid	−0.254	0.790	0.675–0.883	0.750	0.694
Isoleucine	−0.078	0.783	0.679–0.875	0.750	0.666
Leucine	−0.104	0.765	0.646–0.861	0.820	0.638
Oxalic acid	−0.171	0.675	0.552–0.805	0.675	0.611
Ethanolamine	−0.149	0.609	0.492–0.734	0.550	0.666
Alanine	−1.130	0.601	0.471–0.725	0.425	0.805
Caproic acid	−0.089	0.588	0.465–0.705	0.550	0.583
Oleic acid	0.003	0.587	0.448–0.720	0.500	0.722
Lysine	−0.225	0.556	0.435–0.687	0.600	0.611
2-Furoic acid	−0.207	0.551	0.412–0.673	0.650	0.472
Palmitic acid	−4.170	0.544	0.413–0.682	0.675	0.472
Tyramine	−0.220	0.514	0.384–0.640	0.525	0.527
Phenol	−0.465	0.514	0.377–0.640	0.475	0.583

## Data Availability

The raw data are publicly available on the NIH Common Fund’s National Metabolomics Data Repository (NMDR) website, the Metabolomics Workbench, at https://www.metabolomicsworkbench.org/data/DRCCStudySummary.php?Mode=SetupRawDataDownload&StudyID=ST001307 (accessed on 11 March 2023).

## Supplementary Materials

The following supporting information can be downloaded at: https://www.mdpi.com/article/10.3390/metabo13050589/s1, Table S1: The metadata of the analyzed metabolites as extracted from the original study; Table S2: The performance measurements of the permutation test for Leucine and Oxalic acid that has been conducted by Kim et al.
