# Supplementary material for: A Fecal-Microbial-Extracellular-Vesicles-Based Metabolomics Machine Learning Framework and Biomarker Discovery for Predicting Colorectal Cancer Patients
Source: Metabolites. 2023 Apr 25;13(5):589. doi: 10.3390/metabo13050589 (PMC10222315; doi:10.3390/metabo13050589)
Supplement: Supplementary file 1 [file metabolites-13-00589-s001.zip › metabolites-2267459-supplementary.pdf]

Table S1. The metadata of the analyzed metabolites as extracted from the original study by Kim et al.

Metabolite Name	RefMet Name/Standardized Name*	WorkBench Metabolite_ID	Retention time / index	m/z ratio	Level of MSI	Exact mass molecule	Similarity	Reverse	Probability	Chemical Formula
Alanine	Alanine	ME305315	291.15	116	2	89.0932	848	848	6235	$C_3H_7NO_2$
Aminoisobutyric acid	Aminoisobutyric acid	ME305316	444.25	174	2	103.1198	834	845	6732	$C_4H_9NO_2$
Butanoic acid	Butyric acid	ME305317	320	145	2	88.1051	851	870	3894	$C_4H_8O_2$
Ethanolamine	Ethanolamine	ME305318	365.25	174	2	61.0831	742	866	4353	C_2H_7NO
Furoic acid	2-Furoic acid	ME305319	306.2	189	2	112.0835	737	858	6264	$C_5H_4O_3$
Hexanoic acid	Caproic acid	ME305320	275.8	173	2	116.1583	861	878	9200	$C_6H_{12}O_2$
Isoleucine	Isoleucine	ME305321	325.2	86	2	131.1729	737	739	6213	$C_6H_{13}NO_2$
Leucine	Leucine	ME305322	315.15	86	2	131.1729	777	845	6243	$C_6H_{13}NO_2$
Lysine	Lysine	ME305323	599.65	174	2	146.19	712	717	6382	$C_6H_{14}N_2O_2$
Oleic acid	Oleic acid	ME305324	679.35	129	2	282.4614	723	725	6091	$C_{18}H_{34}O_2$
Oxalic acid	Oxalic acid	ME305325	506.55	116	2	90.0349	709	854	4350	$C_2H_2O_4$
Palmitic acid	Palmitic acid	ME305326	631.8	117	2	256.4241	899	899	6134	$C_{16}H_{32}O_2$
Phenol	Phenol	ME305327	953.35	441	2	94.1112	774	774	9579	C_6H_6O
Succinic acid	Succinic acid	ME305328	453.35	233	2	118.088	876	896	4964	$C_4H_6O_4$
Tyramine	Tyramine	ME305329	745.25	174	2	137.179	877	889	4563	$C_8H_{11}NO$

Table S2. The performance measurements of the permutation test for Leucine and Oxalic acid that has been conducted by Kim et al.

Amino Acid	Parameter	p-value	Training set				Test set			
			Sensitivity	Specificity	Accuracy	AUC	Sensitivity	Specificity	Accuracy	AUC
Leucine	0.0000005	0.000	0.86	0.94	0.91	0.94 (CI: 0.92 - 0.96)	0.80	1.00	0.87	0.92 (CI: 0.84 - 1.00)
Oxalic acid	0.0000047	0.000								
